# Complete Blood Count Alterations Prior to the Diagnosis of Colorectal Cancer May Help in the Detection of Synchronous Liver Metastases

**DOI:** 10.3390/jcm12206540

**Published:** 2023-10-15

**Authors:** Rafael J. Sala, John Ery, David Cuesta-Peredo, Vicente Muedra, Vicent Rodilla

**Affiliations:** 1 Department of General and Digestive Surgery, La Ribera University Hospital, 46600 Alzira, Spain; raf.sala.ce@ceindo.ceu.es; 2 Department of Medicine and Surgery, Faculty of Health Sciences, CEU Cardenal Herrera University, CEU Universities, C/Santiago Ramón y Cajal, s/n., Alfara del Patriarca, 46115 Valencia, Spain; vicente.muedra@uchceu.es; 3 RiskLab, ETH Zürich, 8092 Zürich, Switzerland; john.ery@math.ethz.ch; 4 Department of Quality Management, La Ribera University Hospital, 46600 Alzira, Spain; cuesta_davper@gva.es; 5 Department of Anesthesiology, Critical Care and Pain Therapy, La Ribera University Hospital, 46600 Alzira, Spain; 6 Department of Pharmacy, Faculty of Health Sciences, CEU Cardenal Herrera University, CEU Universities, C/Santiago Ramón y Cajal, s/n., Alfara del Patriarca, 46115 Valencia, Spain

**Keywords:** colorectal cancer, synchronous liver metastases, complete blood count, predictive models

## Abstract

**Background and Aims**: Colorectal cancer (CRC) represents 10% of all cancers worldwide with the highest incidence in developed countries; its incidence is also increasing in middle- and low-income countries. Population screening programs facilitate early diagnosis of the disease. When the diagnosis is carried out in advanced stages, approximately 80% of patients with liver metastases (LM) are considered unresectable at the time of diagnosis. In our study, variations in blood counts prior to CRC diagnosis were analyzed to assess whether they could be useful in identifying smaller, more manageable metastases at earlier stages for more effective treatment. **Methods**: A study was carried out using complete blood counts (CBCs) from CRC patients, obtained from primary health centers and the La Ribera University Hospital within La Ribera Health Department, Valencian Community, Spain, between July 2012 and September 2020. Data from CRC patients who presented synchronous liver metastasis (CRLM) were compared with those with CRC without LM at diagnosis (CRC patients). **Results**: Our analysis shows that at least 15 months before CRC diagnosis, a progressive alteration was observed in CBC parameters in both groups. A higher incidence of anemia (*p* < 0.001) was observed among CRLM patients in the three months prior to CRC diagnosis than in CRC patients showing no LM. **Conclusions**: A statistically significant deterioration of CBC was observed in patients with advanced-stage CRC and synchronous or early LM (CRLM) in the three months prior to diagnosis. The primary goal of incorporating CBC variations into predictive models is to identify individuals who are at a greater risk of developing metastatic colon cancer, leading to early diagnosis. Our research improves these models by highlighting a more pronounced and rapid decline in hemoglobin levels among CRLM patients. Identification of metastases at an earlier stage when they are smaller, more manageable, and more amenable to treatment may be a valuable tool to prevent their further progression.

## 1. Introduction

Colorectal cancer (CRC) accounts for 10% of all cancers worldwide, ranking third in terms of incidence, and second in mortality for both sexes [1,2] and it is, at least in the United States, the leading cause of death in men younger than 50 years of age [2]. It has been estimated that 1.9 million new cases of CRC were diagnosed in 2020 worldwide, causing 935,000 deaths [1,3]. Colorectal cancer can be considered as a clear marker of a country’s developmental transition [4] because the incidence rates of CRC are approximately three to four times higher in developed countries [1,5]. In most countries, it increases in parallel to the Human Development Index, probably reflecting the increased prevalence of unhealthy dietary habits and decreased levels of physical activity in those populations [5]. Identification of potentially preventable risk factors such as obesity, lack of physical activity, high red meat intake, alcohol and smoking together with an increasing growing global incidence of this disease, make CRC a global public health challenge [4,6,7]. This malignancy is characterized by its slow progression, typically originating from pre-cancerous growths known as polyps or adenomas, which can remain dormant for years before they transform into malignant tumors. The stage at which colorectal cancer is diagnosed plays a pivotal role in determining patient outcomes and survival rates [7]. For instance, when diagnosed at Stage I, when the cancer is confined to the lining of the bowel, the five-year survival rate can be as high as 93% [8]. Conversely, if the cancer is diagnosed at Stage IV, marked by its spread to other organs, the prognosis becomes significantly worse, with a mere 10% five-year survival rate [8].

The variations in clinical stages at which colorectal cancer (CRC) is diagnosed, partially account for the divergent survival rates documented in the literature, as diagnosis at advanced stages is associated with higher death toll from CRC [9]. CRC metastasis is the major cause of CRC mortality and significantly influences successful treatment [9]. As the liver is the predominant anatomical location for hematogenous metastasis arising from CRC, some strategies have been studied recently, aiming to identify CRC patients who may be at risk of developing liver metastasis (LM) [10,11]. Approximately 20 to 25% of patients diagnosed with colorectal cancer (CRC) are found to be in Stage IV, indicating the presence of metastasis [3]. In subsequent evolution, up to 50% [12] or 60% [13,14] of patients develop liver metastases (LM). More conservative estimates would indicate that 25% of those diagnosed with localized CRC will develop metastasis later [3]. However, 80% [14] to 85% [12] of patients diagnosed with CRC at Stage IV have liver disease considered unresectable at the time of diagnosis [12,14]. In recent years, survival rates for LM patients have increased considerably [12], because of earlier detection, improved surgical methods and the advances in the treatment of liver metastasis [2,12,15]. Tumor size reduction after chemotherapy increases the possibility of surgical salvage [15,16,17], although there is great variability between the different centers, which is why a multidisciplinary approach is recommended [15,18,19,20]. It may be worth mentioning that several nomograms have been developed recently to identify optimal candidates for CRLM resection [21,22] and also to predict survival in colorectal cancer liver metastasis patients after liver resection [23,24].

Complete remission is possible after complete resection, regardless of whether the initial disease was considered unresectable [12]. Survival at 5 and 10 years, ranges from 16% to 74% and 9% to 69%, respectively [12,25]. Even so, most patients remain ineligible for potentially curative surgical resection [12,13].

Early detection of CRC improves survival, reduces mortality, and lessens the burden caused by advanced-stage disease [8,26,27]. However, some studies have shown that early diagnosis, at stages where the disease is still well localized, only occurs in 39% of cases [27]. Screening aims to identify asymptomatic individuals for early detection of the disease, and it is the most effective method for secondary prevention of CRC because it detects silent premalignant and malignant lesions [28]. In developed countries, there seems to be a decline in incidence which has been attributed to a reduction in exposure to risk factors (smoking, excess body weight, alcohol intake, consumption of red and processed meat, low consumption of fruits, vegetables, dietary fiber, and physical inactivity) [2,29,30]. It may also be due to a greater uptake of CRC screening in individuals of 50 years of age or older [2,31]. Despite compelling evidence, a considerable number of eligible individuals choose not to partake in screening programs, resulting in suboptimal compliance rates [26]. Certain studies suggest that an enhancement of the effectiveness of screening can be achieved by selecting high-risk individuals, enabling physicians to dedicate greater attention to them, while emphasizing the benefits of undergoing a colonoscopy [32]. Predictive models based on CBC data can aid in the identification of individuals at a higher risk of CRC for screening [8,33,34,35] and enable targeted screening initiatives.

In this research, we aim to take CBC data one step further, by seeking to find if there are differences between colorectal cancer (CRC) and colorectal liver metastases (CRLM) patients prior to diagnosis of the primary tumor. Our aim was to determine if CBC parameters could be used as a measurement to identify, among all CRC patients, the subgroup of patients with a higher risk for developing LM. Thus, if the group at higher risk for CRLM can be identified, a higher number of patients who are candidates for curative liver resection could be screened and diagnosed earlier. In this framework, the notion of early diagnosis becomes relative, as we are referring to an LM diagnosis occurring somewhat earlier in order to detect them at a stage when the metastatic tumors are smaller, less advanced, more manageable, and easier to treat than those detected at more advanced stages.

## 2. Materials and Methods

### 2.1. Study Design

A preliminary data review to record all CRC patients with diagnosed metastatic lesions using hospital records was conducted for the period 2006–2010, with follow-up until 2020. During this time, 1359 cases of CRC were registered. Of these cases, 279 (20.53%) presented LM at CRC diagnosis or later. However, only 58 cases (20.79%) were eligible for surgical treatment. The limited operability rate prompted us to carry out a further, more complete analysis to explore the potential for identifying LM patients earlier to be able to provide them with the chance of receiving treatment.

To this extent, a retrospective observational study focused on patients diagnosed with CRC was carried out between July 2012 and September 2020 at La Ribera Health Department of the Valencian Community, Spain. La Ribera University Hospital (LRUH) is the reference center for a catchment area of 250,000 people.

All patients with a diagnosis of CRC, including its ICD10 (International Classification of Diseases) codes for colorectal malignancies were selected. Liver metastatic involvement was added as a search criterion.

For comparison purposes, the patients were classified in two groups, those diagnosed with CRC but without LM (CRC) and patients who presented synchronous LM (CRLM). We have defined synchronous metastases as those diagnosed shortly before, at the same time, or up to 3 months after the diagnosis of CRC.

### 2.2. Inclusion and Exclusion Criteria

Patients diagnosed with LM three months after CRC diagnosis were excluded from this study. Patients with other identified causes of concomitant anemia (chronic losses due to erosion in giant hiatal hernia, chronic renal failure, traffic accident and concomitant neoplasms) were also excluded.

### 2.3. Study Variables

This study reviewed CBC data for the patient, whose samples were obtained either from primary care centers or the hospital and were processed in the hospital’s central laboratory. CBC has been classified according to the time of CRC diagnosis, in 3-month periods (T). Thus, T0 includes CBC obtained from the date of CRC diagnosis to 90 days earlier, T1 includes CBC obtained between 91 and up to 180 days prior to CRC diagnosis, T2 from day 181 up to day 270, T3 from day 271 to 360, T4 from day 361 to 450 and T5 CBC obtained more than 451 days before CRC diagnosis.

The selected CBC parameters used in this study were hemoglobin (Hb), Red Blood Cells (RCB), Mean Corpuscular Volume (MCV), Mean Corpuscular hemoglobin (MCH, Mean Corpuscular Hemoglobin Concentration (MCHC), and Red Blood Cell distribution Width (RDW).

### 2.4. Statistical Analysis

Wilcoxon test was used to compare the differences in each CBC parameter between time T0 and the values recorded at Tn (maximal recorded value in the 450 days prior to diagnosis). Welch’s *t*-test (an adaptation of the Student *t*-test for heteroscedastic data) was used to compare mean values in both groups, for each T-period and each CBC parameter. A comparison was initially carried out with each of the periods. The periods with the highest number of records were T5 (data 15 months prior to diagnosis), and T0, (data from the three months prior to the diagnosis of CRC). The periods with the lowest number of records were T4 to T1; for this reason, the analysis was also performed by grouping the central periods with the lowest number of data (T5, T4 + T3, T2 + T1, and T0). For the comparison between the new periods, Welch’s *t*-test has been used. A linear regression with the six T-periods (T0 to T5) as independent variables and the CBC parameters as dependent variables for two groups, CRLM patients and CRC patients, was also carried out. In addition, the differences in the means of the differences have been compared. The degree of change between the variables studied in the CBC has been defined as Delta (Δ), which would allow us to compare the evolution of the values between periods. Thus, for example, (ΔT1 − T0) is the difference in Hb values between the periods 91–180 days (T1) and 0–90 days (T0). All statistical analysis has been carried out with the statistical package R (version 4.1.2).

## 3. Results

**Figure 1:**
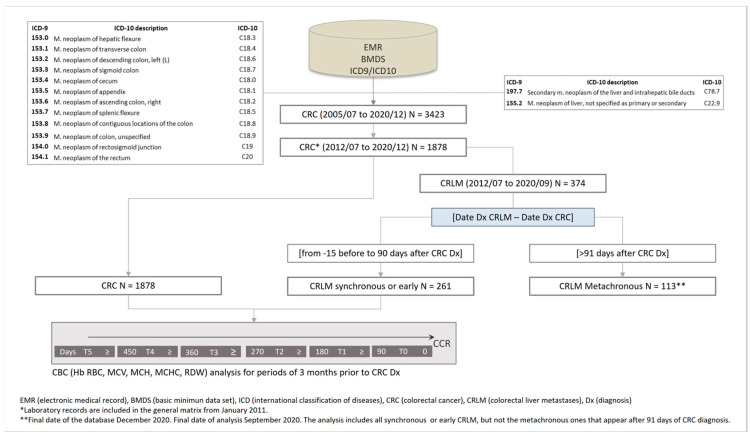
Flowchart illustrating the patient selection procedure used in the study.

Between July 2012 and September 2020, 1878 cases of CRC were registered in this study. The flowchart illustrates the sequence used in patient selection (Figure 1).

Of these cases, 743 (39.56%) were women and 1135 (60.43%) were men. Liver metastasis (LM) was recorded in 374 patients, among which 261 (13.9%) were synchronous.

### 3.1. Prevalence of Anemia in the Study Population

**Figure 2:**
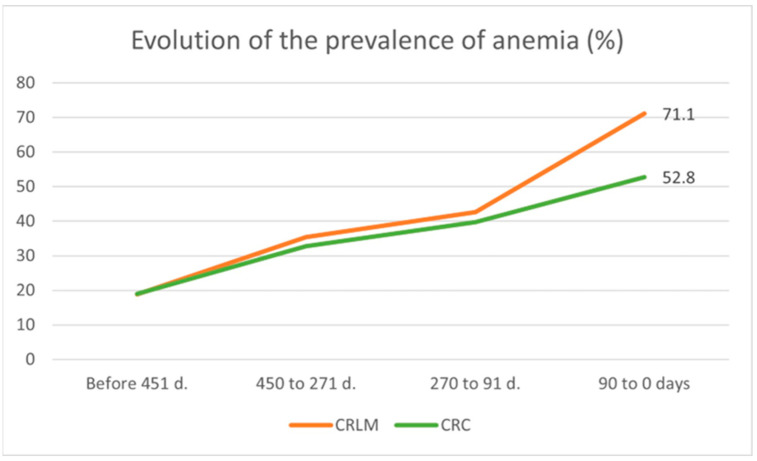
Timeframe evolution of anemia prevalence in CRC (without LM) and CRLM patients at diagnosis.

This study found that prevalence of anemia, which in the adult population has been defined according to the World Health Organization (WHO) criteria as hemoglobin (Hb) values below the lower limits [36], was seen to increase as the time of CRC diagnosis approached. Fifteen months before diagnosis (T5), the prevalence of anemia was 19% for both groups with similar data both for males and females (Table 1). However, three months prior to diagnosis (T0), there was a difference between the two groups (Table 1): up to 71.1% anemia in the liver metastasis group versus 52.8% in the CRC group (Figure 2).

### 3.2. Evolution of the CBC up to CRC Diagnosis

Our study found significant differences in CBC parameters between CRC and CRLM patients when comparing T0 vs. Tn (Table 2). These differences were also present for hemoglobin (HB) and RBC when data were split for gender and the analysis was carried out for men and women. For all parameters, except RDW, there was a sharper drop in CRLM than in CRC. The contrary was seen for RDW, for which a further increase in values can be seen in CRLM than in CRC patients.

To analyze the evolution of CBC up to diagnosis, a linear regression analysis was carried out for both groups and both sexes. The analysis revealed a progressive reduction in Hb, RBC, MCV, MCH and MCHC values, while RDW values increased. This progressive alteration could have been detected 15 months (T5) before diagnosis and as the time of diagnosis (T0) approached (Table 3).

The regression analysis revealed that for all CBC parameters and for both groups, the intercept estimates (the value of the dependent variable when the independent variable is equal to zero) were statistically significant (*p* < 0.001) (Table 3). This means that the value of the intercept is significantly different from zero. Additionally, the slope coefficients relative to time of CRC diagnosis were also significant (Table 3). This means that there is a significant relationship between time and those CBC parameters analyzed.

**Figure 3:**
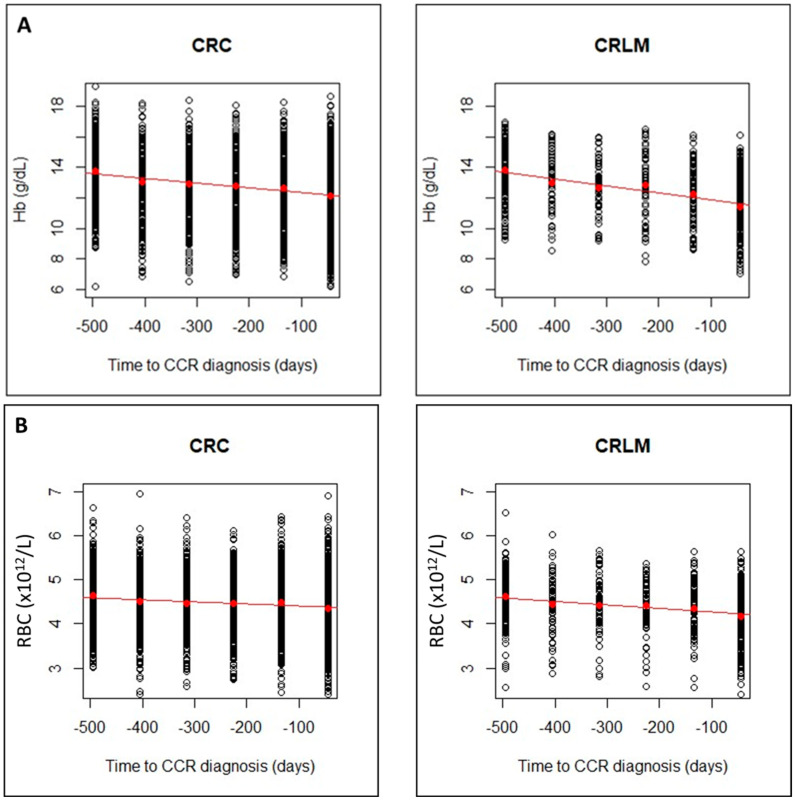

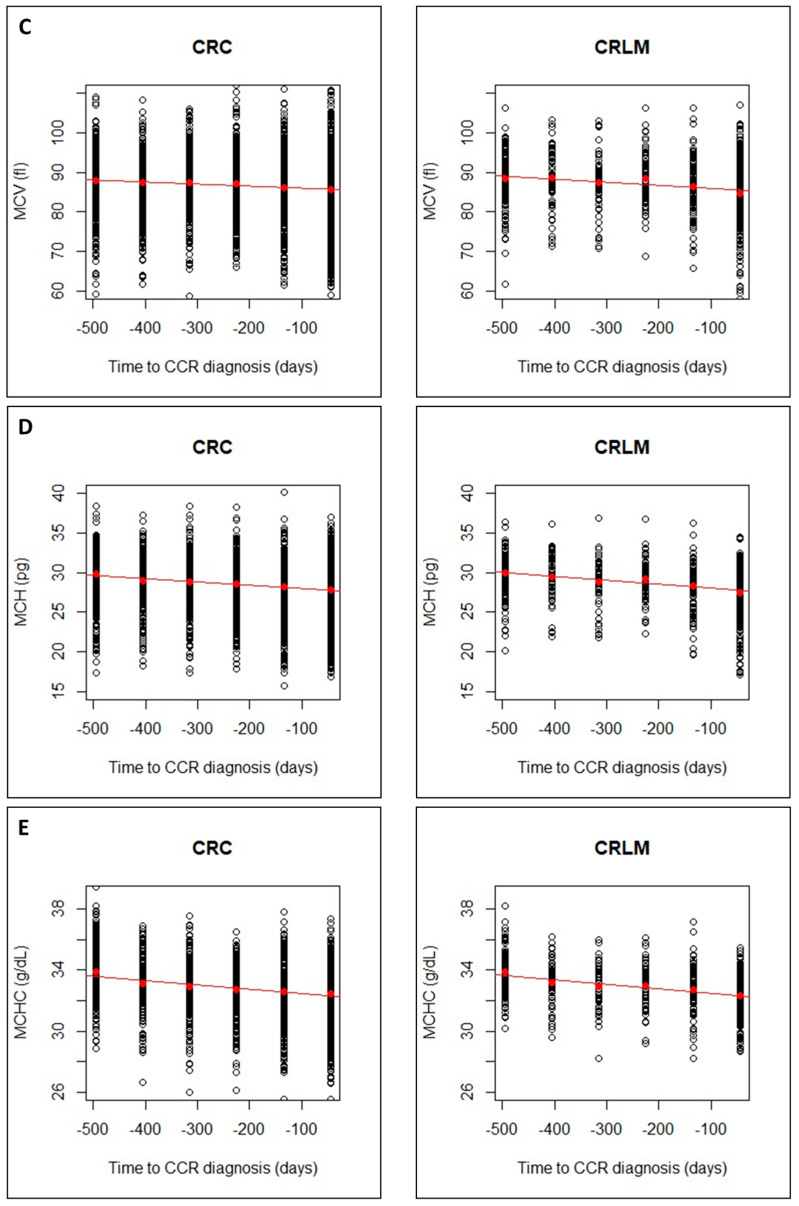

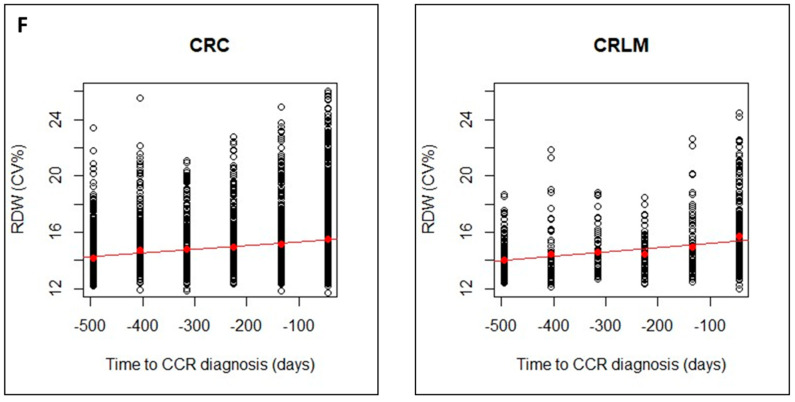
Evolution of CBC values (**A**), hemoglobin (Hb); (**B**), Red Blood Cells (RBCs); (**C**), Mean Corpuscular Volume (MCV)), in CRC and CRLM patients, from 15 months prior diagnosis to the time of diagnosis. Evolution of CBC values (**D**), Mean Corpuscular hemoglobin (MCH; (**E**), Mean Corpuscular Hemoglobin Concentration (MCHC); and (**F**), Red Blood Cell distribution Width (RDW)), in CRC and CRLM patients, from 15 months prior diagnosis to the time of diagnosis. The red lines correspond in each case, to the fitted linear regression line with respect to the means in each time interval (red dots).

The regression lines showed a faster decrease for CRLM group than for CRC group, with higher slopes in absolute value. This can be seen for Hb, RBC and MCV (Figure 3A–C) and for MCH, MCHC (Figure 3D,E) and an increase in RDW (Figure 3F). The linear model can be considered valid for all CBC parameters, but the steeper slope in CRLM, would indicate a greater alteration in these later patients.

### 3.3. Differences in the CBC before the Time of CRC Diagnosis

To analyze this steeper slope in the CRLM group, our study compared the evolution of the means of CBC parameters between both groups using Welch’s *t*-test. Statistically significant differences were observed between both groups for some parameters: T1 for Hb (*p* = 0.027) and RBC (*p* = 0.009), and T0 for Hb (*p* < 0.001), RBC (*p* < 0.001), MCH (*p* = 0.038) and MCHC (*p* = 0.049) (Table 4). This means that when comparing the deterioration that occurs in both groups from 6 months prior to diagnosis (T1 and T0), there is a greater alteration of Hb and RBC in the CRLM group.

To verify the results, data from time periods T4 to T1 were grouped into T4 + T3 and T2 + T1 and analyzed (Table 4).

### 3.4. Comparing the Means of the Differences

Our study has found statistically significant differences between the two groups in the means of hemoglobin levels between two time periods, ΔHbT1 − T0, (*p* < 0.001, Welch’s *t*-test). Comparisons for the remaining blood parameters also produced statistically significant values (Table 4).

In addition to comparing the means of the CBC parameters between the two groups, we have also compared the means of the differences in relation to time. In other words, we have looked at how the change in CBC parameters over time differed between the two groups. Our results show that there were statistically significant differences in these changes between CRC and CRLM patients throughout the course of the study.

Our findings show that the changes in CBC are more pronounced in the CRLM group compared to the CRC group in the 3 months prior to diagnosis. This observation was reinforced by Welch’s *t*-test, which showed that the change in CBC parameters between these two time periods was significantly different between the two groups. ΔT1 − T0 vs. ΔT2 − T1 for Hb, RBC and MCH (*p* < 0.001), MCV (*p* = 0.001), MCHC and RDW (*p* = 0.01).

Furthermore, when comparing the relative differences in CBC changes between CRLM and CRC patients in the three months prior to diagnosis, a greater statistical significance was observed with a smaller *p*-value than when comparing the absolute differences. This means that when considering the extent to which CBC parameters changed in relation to their initial values, the differences between the two groups were even greater (Table 4).

## 4. Discussion

This study found that about 20% of CRC cases had CRLM, which is lower than the 50% reported in other studies [12,13,14,15]. However, some population-based studies have reported similar incidence rates [37,38].

CRC is a type of cancer with metastatic organotropism with secondary tumors showing up in liver, lungs, and the peritoneum more frequently than in other organs like brain or, bone for instance [39,40]. In the patients of this study, most metastasis were detected in liver (21.5%), lungs, pleura, and mediastinum (14.8%) and peritoneum (10.4%). We have detected metastasis in other tissues with a much lower frequency: bone (3.9%), brain and central nervous system (1.7%), ovaries (1.6%), kidney and urinary tracts (1.1%), adrenal glands (1.1%) and skin (0.7%). We limited the data analysis to the liver as it is the most frequently affected organ with metastasis from primary colorectal tumors.

It is noteworthy that in our preliminary assessment we found that 79.21% of mostly synchronous CRLM were not eligible for surgery. This represents 16.26% of all CRC cases. These values are in line with those reported in previous studies [12,13,14,15], which suggests that it is imperative that we find and implement strategies aimed at reducing these rates.

Metachronous metastases appear after the diagnosis and treatment of the primary tumor [41,42] and as these patients are already under the care of an oncological team, they may receive better treatment options. The challenge arises when the disease shows up at a late stage with synchronous metastatic lesions.

However, the definition of synchronous metastases is not consistent across studies [41,42,43,44]. Some authors consider the metastases detected at the time of diagnosis of the primary tumor as synchronous, while others include those LM detected up to the time of surgery [41,42]. However, current recommendation is to define synchronous metastasis as those detected before or at the time of CRC diagnosis [44]. Synchronous liver metastases have also been related to those with a disease-free interval (DFI) ≤ 1 due to a higher risk of recurrence [43,44].

There are different theories regarding the occurrence of metachronous metastases [37], and there is also debate on the definition of the time that must elapse to consider a metastasis as metachronous. However, using the time of diagnosis or intervention on the primary tumor as a standardized cut-off point to define synchronous/metachronous detection is semantically correct [37,44]. The mechanisms of invasion and metastasis are well described in the work of Pretzsch and coworkers [45]. Nesting of circulating tumor cells (CTCs) in a suitable niche, lying dormant until a later time more conducive to their development or with a more permissive immune status, may explain the time lag in the appearance of metachronous lesions. In our study, we included liver metastases diagnosed before and at the time of CRC diagnosis, and up to 3 months after diagnosis. In addition, this period was also chosen because it is the time during which an active metastasis can double in size or become detectable [46,47,48,49,50]. A mean tumor size with a doubling time of 90 days has been observed in liver metastasis secondary to CRC [51]. To estimate the average doubling times of metastases some studies have assessed serial radiological images [46], measurements of the CEA in serum and its doubling time [47], and mutations of different tumor stages in combination with clinical observations [48,49,50]. The results of these studies showed a range of 2 to 4 months in doubling time. For this reason, our decision was to segment the time before diagnosis into 3-month periods.

A systematic review of ten studies aimed at increasing treatability in CRLM patients reported that complete resection was achieved in up to 22.6% of cases after conversion chemotherapy, even with minimal responses [52]. It is reasonable to assume that lesions that shrink with chemotherapy were initially smaller in size. Our study aimed to retrospectively explore laboratory values to detect CRLM cases at a point where treatment would be more feasible. Currently, much research is dedicated to predicting the onset of CRC by using clinical parameters and developing predictive models based on a risk score [53]. Evidencing an increased risk of CRC justifies additional tests such as fecal occult blood test (FOBT), endoscopy, or CT colonography [8,26,32,33,34,35].

The association between anemia and CRC has been previously described [54], with a prevalence between 40 and 60% [55,56,57]. In our preliminary study, when CBC data between CRC patients and those diagnosed with metachronous metastasis were compared, no significant differences were observed. On the contrary, small variations in CBC were observed between CRC patients and those with synchronous metastasis. Hence, the group of patients with metachronous metastasis was excluded from the study. In our study, we found that a higher anemia prevalence of up to 71.1% was observed in the CRLM group compared to 52.8% in the CRC group in the three months prior to CRC diagnosis (Table 1). The etiology of anemia is diverse, and its Positive Predictive Value (PPV) for CRC in primary care is only 1–2% [58,59]. In addition, even though 60% of patients with iron deficiency anemia do not have a related cause, endoscopic exploration would be justified as they are at higher risk of suffering CRC [60].

Before the onset of anemia, there is a silent period. Patients diagnosed with CRC in the asymptomatic phase have less advanced tumors, less undifferentiated tumors, and longer survival. When the patients’ manifest symptoms, the duration of these symptoms such as blood loss and anemia do not correlate with tumor stage and prognosis. A brief history of symptoms does not imply a better prognosis [57,61]. Therefore, the ultimate goal of physicians involved should be to prevent progression from the subclinical to the detectable disease.

Although different studies have shown that serial hemoglobin determinations have limited effectiveness as a diagnostic tool for early tumors, they have successfully identified a significant and a progressive decrease in Hb levels in CRC cases over a period of 3 to 4 years before diagnosis These findings reveal the potential of using such information to advance the subclinical detection of cancer and improve prognosis [62]. Several groups have developed machine learning models based on discrete variations in CBC, age, and sex, which have become capable of identifying individuals with up to a 20-fold increased risk of having occult CRC [32,35]. These models have even suggested the possibility of a more efficient detection of CRC in large populations [34]. However, a large meta-analysis criticizing predictive models has suggested that CBC-based studies could enhance the gold standard for colorectal cancer screening. However, the same study also highlights the importance that predictive models based on colorectal cancer symptoms may need further critical testing [8]. At the same time, a model based on CBC and demographic data was validated to identify patients at high risk of CRC. The algorithm identified 3% of the population at high risk, suggesting that further investigation was required. In the following 6 months, 35% of those patients were diagnosed with CRC [27]. Another study observed a significant correlation between very advanced CRC, compared with locally advanced, in terms of anemia, tumor size and presentation of any symptoms before diagnosis [63]. The study by Li et al. consolidates the usefulness of time trends in Hb prior to diagnosis in CRC and questions whether the use of these algorithms could influence the distribution of CRC stages [59]. All these studies have compared patients with CRC and controls without this pathology.

In the present study, and building upon previously validated comparisons, we conducted a comparative analysis between CRLM and CRC patients which to our knowledge has never been conducted. As in the CRC risk prediction models, it was possible to verify a significant progressive alteration (*p* < 0.001) in the CBC in the two groups from 15 months prior to the moment of CRC diagnosis, decreasing for Hb, RBC, MCH, MCV and, MCHC and increasing for RDW (Table 3; Figure 2 and Figure 3). In addition, a greater alteration (*p* < 0.001) in Hb and RBC levels was observed in the CRLM patient group in the three months prior to CRC diagnosis (Table 4).

The significance of these observations highlights the potential of predictive models in achieving early diagnosis of CRC as they provide complementary information for the development of decision trees using Artificial Intelligence in the early detection strategies within Primary Care [64]. The progressive deterioration of hematic parameters in all CRC cases, in our study both CRC and CRLM groups, should serve as a warning to complete the differential diagnosis of anemia and rule out the presence of an underlying CRC. Our contribution is the discovery of a greater deterioration in the three months prior to diagnosis in those colorectal cancer patients with synchronous LM (CRLM). If we reach diagnosis at this stage of progression, a point at which CRLM is already present, the concept of early diagnosis becomes relative because we could only refer to it as somewhat earlier diagnosis. The aim is not to prevent the development of metastases but rather to detect them at an earlier stage when they are smaller, in less advanced stages and therefore easier to treat. Predictive models can facilitate “pre-staging” and improve the “treatability” of these patients. When it comes to metastases, a mere three months can determine whether a patient is eligible for surgery or not. The ultimate goal of these type of studies is to achieve such an early diagnosis that would prevent the occurrence of CRLM altogether.

Some studies have indicated that preoperative serum CEA level plays a significant role in the prognosis of CRC patients as an independent risk factor for prognosis [21,65,66,67]. Thus, in the recent scientific literature, the preoperative serum CEA level has emerged as a key prognostic marker [65]. Its significance lies in its capacity to offer valuable insights into the expected course of CRC and its potential impact on patient survival and recovery. The robust and consistent findings from these investigations have led to a growing consensus among medical professionals regarding the pivotal role of CEA and have firmly established CEA as a potent independent risk factor, shedding light on its critical role in predicting the clinical outcome of CRC patients [66,67]

The *Septin 9* gene, encoding GTP-binding proteins, holds a pivotal role in the initiation and advancement of colorectal cancer (CRC), as indicated by previous research [68]. In nearly all CRC tissues, methylated *Septin 9 DNA* (*mSEPT9*) has been detected [69,70]. Recent investigations have illuminated the potential of *mSEPT9* as a highly promising biomarker for CRC detection. Current research underscores the possibility of utilizing *mSEPT9* in peripheral blood not only to assess the pathological stages of CRC but also to use it as a molecular biological indicator to gauge prognosis, recurrence, and metastasis in CRC patients [71,72,73].

However, screening with these indicator tests (CEA, *mSEPT9* and others [74]) cannot be used for the general population because of costs. Therefore, it would perhaps be plausible to detect possible candidates for CRC and CRLM through a simple and inexpensive CBC analysis, to identify those candidates needing screening for molecular markers, with higher sensitivity and specificity for CRLM.

One of the limitations of this study is that it is retrospective. The database used lacks homogeneity in its CBC records, as they were arbitrarily conducted and mostly times and parameters were based on the criteria of the general practitioners and the care requirements of each patient, obviously before CRC diagnosis. Consequently, there are missing data in the database, resulting in data heterogeneity between time periods. Further studies to demonstrate the validity of our observations would imply designing an appropriate prospective cohort study including both CRC and CRLM as well as non-CRC patients (controls) with adequate time-points for CBC determination.

We enter into a complex and largely uncharted territory within clinical practice as we have a poor understanding of how these CBC component levels change over time. It is also not known how many repeated CBC measurements might be necessary to reliably identify the presence of colorectal cancer and concomitant liver metastasis, particularly in its early stages. Addressing these questions and further exploring the intricate relationship between CBC components in the detection and diagnosis of colorectal cancer with synchronous liver metastasis holds, in our view, great promise for refining early detection strategies. This research may lead to the development of more precise and effective diagnostic protocols, ultimately offering individuals at risk of colorectal cancer in general and those with underlying CRLM a better chance of timely intervention and improved long-term outcomes.

## 5. Conclusions

Approximately 15 months before diagnosis, a slight deterioration of CBC can be observed in both groups of patients, those who do not develop LM (CRC) and those who do (CRLM). More important, we found a statistically significant deterioration in CBC and a higher incidence of anemia in CRLM patients in the three months leading up to CRC diagnosis. Our study sheds light on the subtle, yet significant changes that occur in the months preceding diagnosis. Therefore, these findings have the potential to offer valuable complementary information for the development of more complete screening tests. It could also lead to the development and use of decision trees coupled with Artificial Intelligence for early detection strategies within Primary Care. 

## Figures and Tables

**Table 1:** Evolution of the prevalence of anemia in patients with CRC, comparing both groups (CRC and CRLM at diagnosis), as the time of diagnosis approaches. Columns represent the total number of individuals (N) and the number of patients with anemia, together with the percentage of anemia for both sexes and each group (CRLM and CRC)

		Before 451 Days			450 to 271 Days			270 to 91 Days			90 to 0 Days		
		N	Anemia	%	N	Anemia	%	N	Anemia	%	N	Anemia	%
CRLM	Women	68	14	20.6	38	10	26.3	48	16	33.3	81	61	75.3
	Men	134	24	17.9	75	30	40.0	93	44	47.3	161	111	68.9
	Total	202	38	18.8	113	40	35.4	141	60	42.6	242	172	71.1
CRC	Women	470	89	18.9	275	82	29.8	341	124	36.4	483	245	50.7
	Men	618	118	19.1	375	131	34.9	481	202	42.0	689	374	54.3
	Total	1088	207	19.0	650	213	32.8	822	326	39.7	1172	619	52.8

**Table 2:** Average values ± SD for CBC in CRC and CRLM patients at T0 (0–90 days prior to diagnosis) and Tn (maximal recorded value in the 450 days prior to diagnosis) and their reference values.

	CRC without LM	CRLM	Reference Values
Hb_T0	11.72 ± 1.93 (Men) 12.41 ± 2.42 (Women)	11.03 ± 1.61 (Men) 11.56 ± 2.14 (Women)	13.0–17.5 g/dL (Men) 12.0–16.5 g/dL (Women)
Hb_Tn	13.31 ± 1.27 (Women) 14.60 ± 1.60 (Women)	13.15 ± 1.27 (Women) 14.39 ± 1.49 (Women)	
RBC_T0	4.24 ± 0.54 (Men) 4.44 ± 0.67 (Women)	4.01 ± 0.53 (Men) 4.25 ± 0.62 (Women)	4.5–5.9 × 10^12^/L (Men) 4.1–5.2 × 10^12^/L (Women)
RBC_Tn	4.49 ± 0.46 (Women) 4.90 ± 0.49 (Women)	4.49 ± 0.46 (Women) 4.83 ± 0.50 (Women)	
MCV_T0	85.54 ± 8.33	84.64 ± 8.71	80–99 fl
MCV_Tn	89.34 ± 6.25	89.50 ± 6.21	
MCH_T0	27.85 ± 3.53	27.41 ± 3.53	26–34 pg
MCH_Tn	30.08 ± 2.32	30.14 ± 2.34	
MCHC_T0	32.48 ± 1.64	32.30 ± 1.49	31–37 g/dL
MCHC_Tn	30.08 ± 2.32	34.08 ± 1.59	
RDW_T0	15.53 ± 2.45	15.74 ± 2.36	11.5–15.0 CV%
RDW_Tn	15.10 ± 2.04	14.82 ± 1.76	

**Table 3:** Linear regression of CBC parameters with time as an independent variable. Highlighted in bold are those instances in which the probability found was greater than 0.05 (*p* > 0.05)

Linear Regression		From T5 (−451 Days)-----------------------------------to T0 (−90 to 0 Days)					
			R^2^	Intercept	*p*-Value	Time to CRC Diagnosis	*p*-Value
Hb	Women	CRLM	0.69	11.30	<0.001	−0.0033	0.0397
		CRC	0.89	11.70	<0.001	−0.0025	0.0043
	Men	CRLM	0.88	11.50	<0.001	−0.0052	0.0054
		CRC	0.84	12.27	<0.001	−0.0035	0.0103
RBC	Women	CRLM	0.48	4.07	<0.001	−0.0006	**0.1290**
		CRC	0.90	4.25	<0.001	−0.0004	0.0039
	Men	CRLM	0.95	4.24	<0.001	−0.0010	0.0110
		CRC	0.61	4.45	<0.001	−0.0005	**0.0682**
MCV	Women	CRLM	0.58	86.37	<0.001	−0.0066	**0.0768**
		CRC	0.49	85.43	<0.001	−0.0036	**0.1200**
	Men	CRLM	0.62	84.60	<0.001	−0.0084	**0.0625**
		CRC	0.93	85.66	<0.001	−0.0057	0.0018
MCH	Women	CRLM	0.77	27.94	<0.001	−0.0039	0.0214
		CRC	0.87	27.61	<0.001	−0.0031	0.0062
	Men	CRLM	0.79	27.24	<0.001	−0.0056	0.0184
		CRC	0.94	27.63	<0.001	−0.0045	0.0016
MCHC	Women	CRLM	0.80	32.30	<0.001	−0.0021	0.0164
		CRC	0.87	37.27	<0.001	−0.0023	0.0066
	Men	CRLM	0.80	32.13	<0.001	−0.0034	0.0154
		CRC	0.85	32.16	<0.001	−0.0031	0.0086
RDW	Women	CRLM	0.35	15.08	<0.001	0.0024	**0.218**
		CRC	0.87	15.51	<0.001	0.0025	0.007
	Men	CRLM	0.88	15.79	<0.001	0.0036	0.0052
		CRC	0.89	15.66	<0.001	0.0028	0.0048

**Table 4:** Summary of two-sample statistical analysis between the different time periods T0 to T5 from 90 days before the time of diagnosis (T0) to more than 451 days before diagnosis (T5).

	>451 Days	450–361 Days	360–271 Days	270–181 Days	180–91 Days	90–0 Days
Difference in CBC before the time of diagnosis in days						
Welch’s test	T5	T4	T3	T2	T1	T0
Hb	-	-	-	-	0.027	<0.001
RCB	-	-	-	-	0.009	<0.001
MCV	-	-	-	-	-	0.072
MCH	-	-	-	-	-	0.038
MCHC	-	-	-	-	-	0.049
RDW	-	-	-	-	-	-
Difference in CBC matching the data two by two						
Welch’s test	T5–T4		T3–T2		T1–T0	
Hb	-		-		<0.001	
RCB	-		-		<0.001	
MCV	-		-		-	
MCH	-		-		0.078	
MCHC	-		-		-	
RDW	-		-		-	
Differences in the means of differences						
					ΔT1 − T0	
Welch’s test	T5	T4	T3	T2	T1	T0
Hb	-	-	-	-	0.080	<0.001
RCB	-	-	-	-	0.081	0.005
MCV	-	-	-	-	-	0.017
MCH	-	-	-	-	-	0.004
MCHC	-	-	-	-	-	0.022
RDW	-	-	-	-	-	-
				Δ0–90 vs. 91–270 d		
Welch’s test	T5	T4–T3		T2–T1		T0
Hb	-	-		-		<0.001
RCB	-	-		-		<0.001
MCV	-	-		-		0.001
MCH	-	-		-		<0.001
MCHC	-	-		-		0.011
RDW	-	-		-		0.011

## Data Availability

Data were obtained from the Hospital medical records and could be made available upon request to the authors with the permission of the Hospital authorities.
